# Aptamer decorated PDA@magnetic silica microparticles for bacteria purification

**DOI:** 10.1007/s00604-024-06322-3

**Published:** 2024-04-23

**Authors:** Murat Kavruk, Zahra Babaie, Güneş Kibar, Barbaros Çetin, Hasan Yeşilkaya, Yassine Amrani, Ali Doğan Dursun, V. Cengiz Özalp

**Affiliations:** 1 Department of Medical Biology, Faculty of Medicine, İstanbul Aydın University, İstanbul, 34295 Türkiye; 2 Microfluidics & Lab-on-a-chip Research Group, İ.D. Bilkent University, Ankara, 06800 Türkiye; 3 UNAM-National Nanotech, Research Center and Institute Materials Science & Nanotech, İ.D. Bilkent University, Ankara, 06800 Türkiye; 4 Micro Nano Particles (MNP) Research Group, Materials Science and Engineering Department, Adana Alparslan Turkes Science and Technology University, Adana, 01250 Türkiye; 5 Department Respiratory Sciences, University of Leicester, University Road, Leicester, LE1 7RH UK; 6 Department of Physiology, School of Medicine, Atilim University, Ankara, 06830 Türkiye; 7 Department of Medical Biology, School of Medicine, Atilim University, Ankara, 06830 Türkiye

**Keywords:** Biosensor, Aptamer, Aptasensor, SELEX, Superparamagnetic, Microbeads

## Abstract

One significant constraint in the advancement of biosensors is the signal-to-noise ratio, which is adversely affected by the presence of interfering factors such as blood in the sample matrix. In the present investigation, a specific aptamer binding was chosen for its affinity, while exhibiting no binding affinity towards non-target bacterial cells. This selective binding property was leveraged to facilitate the production of magnetic microparticles decorated with aptamers. A novel assay was developed to effectively isolate *S. pneumoniae* from PBS or directly from blood samples using an aptamer with an affinity constant of 72.8 nM. The capture experiments demonstrated efficiencies up to 87% and 66% are achievable for isolating spiked *S. pneumoniae* in 1 mL PBS and blood samples, respectively.

**Graphical abstract:**

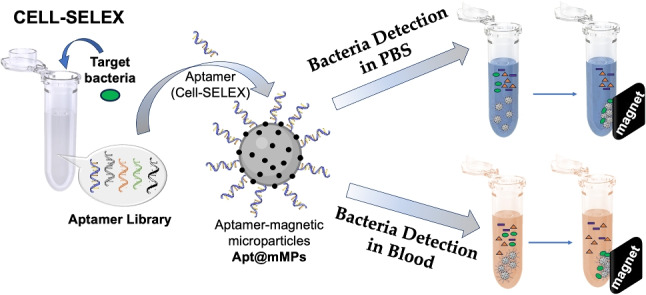

## Introduction

Bacterial infections are one of the significant public health threats with high mortality rates in the world [1]. *Streptococcus pneumoniae* (*S. pneumoniae*), a Gram-positive spherical bacterium, is responsible for a significant proportion of community-acquired pneumonia (CAP) cases in adults, children, and infants, resulting in more than 6 million deaths in 2019 [2]. *S. pneumoniae* is a significant cause of bacteremia, meningitis, upper respiratory tract infections, and otitis media worldwide [3]. Despite the development of a 13-valent pneumococcal conjugate vaccine (PCV), *S. pneumoniae* has high morbidity and mortality rates [4]. Currently, the diagnosis of pneumococcal diseases relies on conventional bacterial culture to isolate microbes from the specimen, which generally takes about 24 to 48 h [5]. The other methods of detection are the PCR-based nucleic acid amplification test (NAAT) and immunochromatography assay (ICA). While these methods are rapid and accurate, NAAT is expensive and requires specific equipment. In ICA, the chance of a misdiagnosis is high, and it has a limited sensitivity [6].

Various strategies have been employed to enhance the selective detection of target bacteria from complex samples [7, 8]. These include utilizing antibody-antigen interactions [9], aptamer binding [10], bacteriophage biological recognition [11], capture using antibiotics or antimicrobial peptides [12], and other interactions [13] involving target recognition. Immunomagnetic bead-based separation is also a widely adopted technique especially in microfluidic devices [14]. This method utilizes magnetic microparticles (mMPs) coated with specific binding ligands that selectively bind to target biomolecules, enabling isolation of the mMPs-complex within the system for further analysis [15, 16]. mMPs possess remarkable qualities such as high magnetic susceptibility, superparamagnetism, and biocompatibility, which distinguish them from alternative methods with microparticles. The magnetic field is employed to capture and isolate mMPs along with the biomolecules they have bound to. Achieving a high recovery rate is crucial for the purification of bacteria cells. The use of mMPs offers advantages such as easy isolation within the system using an external magnetic field, a high surface area-to-volume ratio allowing for ample binding sites, and customization to target different biomolecules [17, 18]. These advantages make separation using mMPs a highly promising, reliable, and versatile option for bacteria detection especially with a high potential to be integrated into microfluidic systems.

Aptamers, which are synthetic DNA or RNA oligonucleotides generated through in vitro selection methods, possess remarkable affinity and specificity for binding to specific molecular targets [19]. The major advantage of aptamers to other affinity molecules is the robust synthetic selection procedure for any target and subsequent supply of machine-synthesized nucleic acid products. Aptamers also offer several other advantages, including precise synthesis, easy modification, high purity, smaller molecular weight (approximately 12–30 kDa), and cost-effectiveness. They also exhibit improved thermo/pH stability, making them suitable for easy storage [20, 21]. Therefore, aptamers have gained significant popularity in recent years as a means of recognizing and binding to analytes [20] and have found applications in many areas including bioanalysis and targeted therapy [22–24]. Aptamers are generated in vitro through a selection method known as systematic evolution of ligands by exponential enrichment (SELEX), which is based on repeated cycles of partitioning and amplification from a random synthetic oligonucleotide library [25]. Recently, a variant of the conventional SELEX method was developed as Cell-SELEX, which uses the whole cell to recognize the targeted pathogen surface by selecting a three-dimensional structure to bind the interaction surfaces [26]. The process of selecting aptamers for bacterial cells mostly relies on whole Cell-SELEX procedure. This method is a modified version of the traditional SELEX strategy, which typically involves using purified target molecules [27]. DNA aptamers acquired by bacterial Cell-SELEX techniques have become increasingly popular as a means of obtaining innovative and cost-efficient affinity agents for biosensing. In the process of Cell-SELEX, DNA aptamers are commonly chosen from extensive collections of randomized single-stranded DNA (ssDNA) libraries, which typically contain a range of 10^12^ to 10^15^ distinct sequences. This selection is achieved by utilizing entire bacterial target cells and separating them through centrifugation. Cell-bound ssDNA sequences are amplified using polymerase chain reaction (PCR) in each cycle of selection, following the same process as standard SELEX procedures. The amplified sequences are then regenerated for the subsequent round of Cell-SELEX. Following several iterations of selection (usually ranging from 6 to 20 rounds), the ultimate enriched ssDNA pool is inserted into plasmids and subsequently subjected to Sanger sequencing or direct next-generation sequencing (NGS) in order to discover potential aptamer candidates [28]. In traditional SELEX procedure, the target molecule is immobilized on a solid surface for separating target-bound and unbound library members [29]. However, Cell-SELEX procedures allow selecting aptamers that interact directly with cell surface components in their native conformation with no prior knowledge of the molecular target. Since the complex components on the cell surface may bias the library towards highly expressed epitopes, several combinatorial strategies have been reported such as precision-SELEX [30] or cross-over SELEX [31]. Recently, both SELEX and Cell-SELEX have been implemented to generate the possible aptamers for different pathogens and their subspecies, such as *Escherichia coli* [32], *Staphylococcus aureus* [33], *Bacillus cereus* [34], *Streptococcus agalactiae* [35], *Rikinella microfusus* [36], and many other medical and food-born pathogenic bacteria [37, 38], to utilize them in biosensing or purification applications.

In this study, we employed the Cell-SELEX technique to generate a new aptamer series targeting specifically *S. pneumoniae* as a whole pathogen in a mixture of non-target bacterial strains of *E. coli*, *S. aureus*, and *L. monocytogenes*. After selecting the aptamer, we designed a biosensing and bioseparation method utilizing in-house synthesized silica mMPs. For this purpose, silica mMPs were synthesized and biomimetically surface modified with polydopamine (PDA@mMPs) to attach the Cell-SELEX aptamer to recognize and purify *S. pneumoniae* from PBS and blood samples. Synthesis of mMPs and successful isolation of *S. pneumoniae* in a batch system is the first step towards the isolation of *S. pneumoniae* on a novel 3D-printed magnetic microfluidic platform [39, 40] for rapid detection [41].

## Materials and method

**Table 1:** SELEX library and PCR primer sequences used during aptamer selection

Name	Sequence
SELEX library	GGCGGCGATGAGGATGAC-N40-ACCACTGCGTGACTGCC
Forward primer	GGCGGCGATGAGGATGAC
Reverse primer	GGCAGTCACGCAGTGGT
NGS forward primer	TCGTCGGCAGCGTCAGATGTGTATA
	AGAGACAGGGCGGCGATGAGGATGAC
NGS reverse primer	GTCTCGTGGGCTCGGAGATGTGTAT
	AAGAGACAGGGCAGTCACGCAGTGGT

**Fig. 1:**
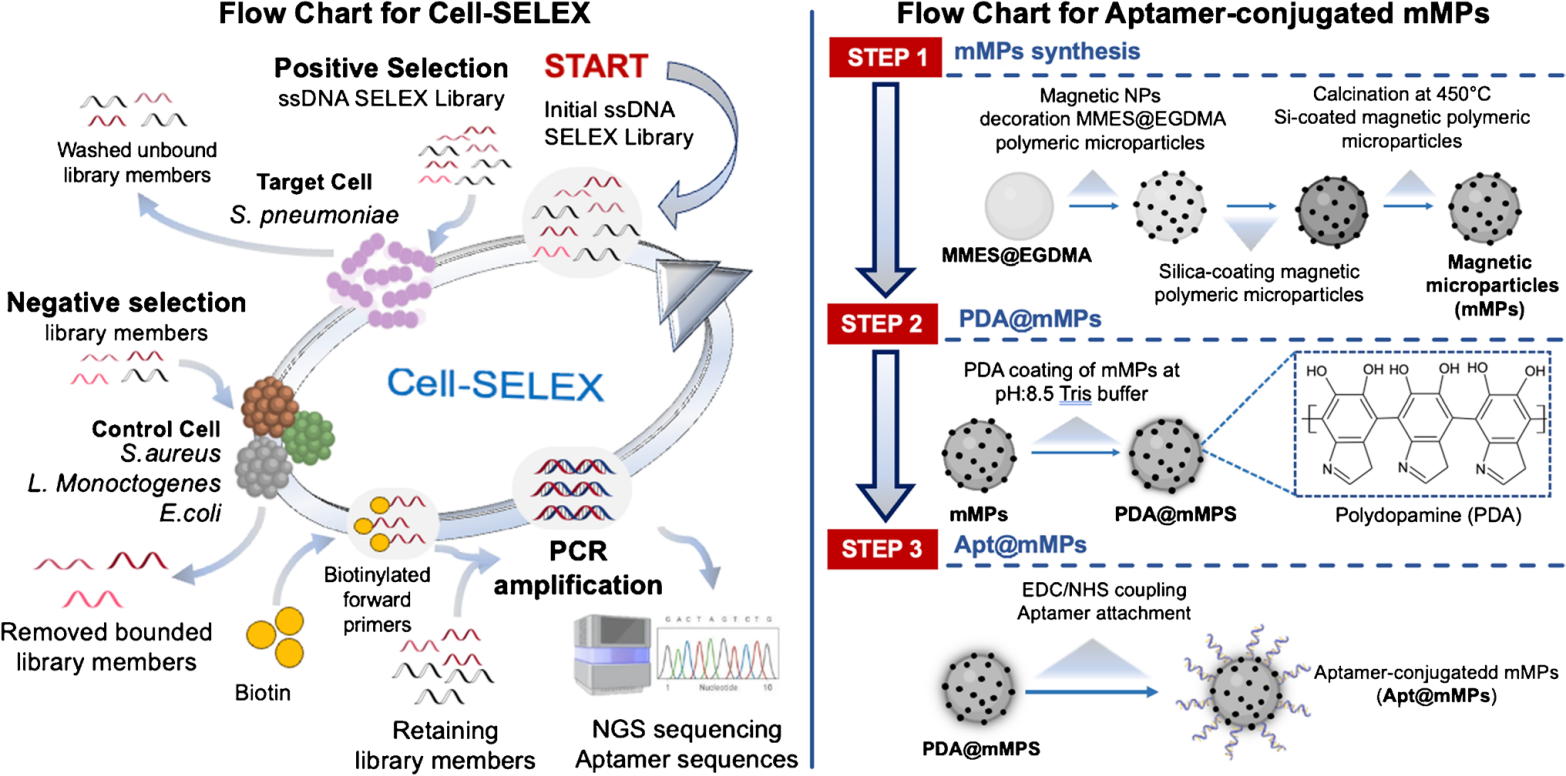
Flow chart for Cell-SELEX and microparticle synthesis

The sequences and primers of the DNA SELEX library employed in this study were from a previously published study [42]. The flow chart of a Cell-SELEX cycle is illustrated in Fig. 1. The library was 79 nucleotides long in total, consisting of a middle section with 40 random nucleotide sequences and fixed primer sites placed at both ends for PCR reactions (please see Table 1). All other chemicals were obtained from commercial companies. A mixture of *S. pneumonia* isolates was used in Cell-SELEX procedure [42]. In negative SELEX experiments, aptamer sequences with high specificity were obtained by using a mixture of non-target bacterial strains consisting of *E. coli*, *S. aureus*, and *L. monocytogenes*. In the second stage, the bacterial mixtures obtained from the previous stage were combined with the DNA library members to enrich the sequences with binding potential. In the third stage, aptamer candidate sequences were determined in the enriched SELEX cycle and analyzed with bioinformatics methods. In the final stage, the oligonucleotides whose sequences were determined were characterized using flow cytometry analysis in affinity experiments performed after fluorescent labeling. The equilibrium binding of an aptamer can be described by a form of Langmuir isotherm as follows [43]:

1 $$\begin{aligned} \left( \frac{q}{q_{m}}\right) = \frac{C}{K+C} \end{aligned}$$

where *C* [nM] is the equilibrium concentration of aptamer, *q* is the amount of aptamer bound to cells, $q_m$ is the maximum amount of aptamer bound to cells, and *K* [nM] is the binding affinity. The amount of aptamer bound to cells were quantified by a fluorescent signal. Therefore, the adsorption model can be written as follows:

2 $$\begin{aligned} \left( \frac{A}{A_{m}}\right) = \left( \frac{q}{q_{m}}\right) = \frac{C}{K+C} \end{aligned}$$

where *A* [a.u.] is the fluorescent signal at specific aptamer concentration and $A_m$ [a.u.] is the fluorescent signal which corresponds to maximum aptamer binding. Nonlinear curve fitting is performed by the built-in function lsqcurvefit, which utilizes the Levenberg-Marquardt algorithm, available in MATLAB^®^. The regression coefficient is defined as follows:

3 $$\begin{aligned} R^2 = 1-\frac{\sum \limits _{j} (A_{j}-{A}_{\text {predicted}})^2}{\sum \limits _{j}(A_{j}-\bar{A})^2} \end{aligned}$$

where $\bar{A}$ is the mean of the $A_{j}$ data.

The SELEX was performed based on previously published methods for bacterial cells [42]. More than 100 serotypes exist for *S. peunomoniae*. In this study, five of the most invasive serotypes were used for aptamer selection (please see Table 2) [44]. Thus, the recognition of serotypes 2, 4, 14, 6B, and 19F were targeted by the selected aptamers. *S. pneumoniae* strains and reference strains of *S. aureus* (ATCC 25923), *E. coli* (ATCC 12435), and *L. monocytogenes* (ATCC 19115) were obtained from glycerol stocks frozen at $-$80 ^∘^C and incubated in BHI (Brain Heart Infusion) at 37 ^∘^C for at least 24 h. The bacterial concentrations were adjusted by turbidity readings at 600 nm to 10^8^ CFU/mL in PBS. The stocks were first checked by growing them in blood agar medium, and then they were grown in BHI broth in stock preparation.

### SELEX cycles

**Table 2:** *S. pneumoniae* serotypes employed during aptamer selection

Isolate	Mutations	Serotype
S244	D39 wildtype	2
S245	TIGR4	4
S247	BHN-78	14
S248	BHN-191	6B
S249	BHN-457	19F

The mixture of *S. pneumoniae* isolates prepared to start the SELEX cycles as seen in Table 2. In short, the combinatorial DNA library, which consists of approximately 10^14^ different sequences, was combined with the bacterial mixture prepared in the first step, and the SELEX cycle of binding and dissociation was applied. While positive selection was made with the target organism (*S. pneumoniae*), negative selection was performed by a mixture of non-target bacteria (*E. coli*, *L. monocytogenes*, and *S. aureus*). After the sequences binding to the library members bound to the target cells were separated by centrifugation (10,000 rpm, 1 min), they were amplified by PCR (95 ^∘^C, 3 min, 35 cycles (95 ^∘^C, 1 min; 61 ^∘^C, 1 min; 72 ^∘^C, 1 min; 72 ^∘^C, 5 min) using 5’-biotinylated forward primers (Table 1), and single-stranded sequences were obtained using magnetic streptavidin-coated particles. The ratio of bound library members to bacteria was evaluated at the end of each cycle. The cycle pool was amplified by PCR with a 5’-fluorescently labelled primer and binding amount to the *S. pneumoniae* mixture was evaluated after each cycle in triplicates. The single-stranded fluorescently labelled PCR products were incubated with target bacteria or non-target bacteria and washed three times with PBS buffer solution, and quantitative analysis of the aptamer sequences remaining on the cell surface was performed by fluorescence spectroscopy.

### Selection of aptamer candidates

NGS is a high-throughput sequencing method used in aptamer selection and has been applied in many aptamer selection studies [28]. PCR products obtained at the end of the final cycle were amplified again with NGS forward and NGS reverse primers (Table 1), and NGS procedure was performed (iSeq100, Illumina). The experimental procedures applied were similar to previous publications except the analysis part [45]. In short, Nextera XT index Kit v2 Set-A (Illumina) was employed to label each sample with index and washed with AMPure, and the amount of PCR products was determined with a spectrophotometer (Omega, Labtech, Germany). All samples were dissolved in a 10 nM mixture and loaded into the iSeq 100 cartridge by adding 5% (v/v) PhiX control v3 (15017666, Illumina) to a final concentration of 35 pM. Sequences were converted to FASTQ file type in the Illumina basespace database, and the results were separated from the fixed primer regions of the adapter and library using the Galaxyuse software. The obtained sequences were divided into groups according to common motif regions according to a MEME SUITE analysis [46]. The resulting aptamer candidate sequences were machine-synthesized with 5’-FAM label, and their binding affinities were calculated using the flow cytometry analysis based on published methods in triplicates [27, 47]. The aptamer and 10^4^ bacterial cells were mixed in PBS buffer and incubated for 30 min with constant agitation. The fluorescence intensity of FAM was measured with flow cytometry in the fluorescein isothiocyanate (FITC) channel (CytoFLEX, Beckman & Coulter, USA) in triplicates. Each sample was analyzed three times, recording 5000 events per measurement. To quantify the binding, we compared the fluorescence signal intensity between the control sample (without aptamers) and various aptamer concentrations. The differences were expressed as a percentage increase relative to the corresponding aptamer concentrations. This analysis was conducted using the CytExpert software (Beckman Coulter, USA). The resulting binding curves were then used to calculate the affinity constants (K) using the Langmuir model as described in Eqs. (1) and (2).

**Table 3:** The aptamer candidates selected in this study

Name	Sequence
Aptamer#1	CCACCATTGAGATTATAG**AAGAATT**CGCGTGAAGGCCGAGG
Aptamer#2	TCTGCCCGCTGACTTGACCCATT**CTTCTA**TAATCTTTACGG
Aptamer#3	GTCGAGAATGACCCCGCCCCCTATACA**TCTCAG**GTGCGTCT
Aptamer#4	CCTCGGCCTT**CACGCG**AATTCTTCTATAATCTCAATGGTGG

The bold highlighted nucleotides are the motif sequences

### Preparation of aptamer-conjugated mMPs

The preparation of aptamer-conjugated mMPs was performed mainly in three steps, namely (i) microparticle synthesis, (ii) biomimetic surface modification, and (iii) aptamer binding, as summarized in Fig. 1.

#### Microparticle synthesis

The synthesis of magnetic nanoparticle-decorated silica microparticles followed a procedure similar to that outlined in the literature [40]. As described in Fig. 1, the synthesis of mMPs (STEP 1) occurred in three distinct steps. Initially, MMES-co-EGDMA compounds were synthesized via multistage template polymerization [48]. In this process, Poly(GMA) seed particles, synthesized through dispersion polymerization, served as templates [49]. Subsequently, the MMES-co-EGDMA microparticles underwent magnetization, as outlined in previous literature [50]. Following the magnetization of the polymeric particles, silica particles were fabricated onto them [51].

#### Biomimetic surface modification of mMPs

A bio-based green route for particle surface modification was implemented to obtain functional groups on superparamagnetic monodisperse microparticles [52]. The mimic of mussel adhesive protein dopamine was employed to obtain self-assembly polymerized polydopamine (PDA) on superparamagnetic silica microparticles (PDA@mMPs). Basically, 0.25 g of the magnetic particle and DOPA HCl (2 mg/mL) were dispersed in 10 mL of pH 8.5 Tris buffer (please see Step 2 in Fig. 1). The mixture was mechanically stirred in the dark hood for 6 h. Then, the particles were collected *via* a strong natural magnet and washed with deionized water several times. The particles were dried at 50 ^∘^C in a vacuum oven for further modifications.

The morphological structure of the mMPs was characterized *via* scanning electron microscope (SEM, Quanta 450 SEM; Akishima, Tokyo, Japan) and energy-dispersive X-ray spectroscopy (EDX, Quanta 450 SEM; Akishima, Tokyo, Japan). The chemical structure of the particles was determined using Fourier-transform infrared spectroscopy. The magnetic properties were analyzed by vibrating sample magnetometer (VSM-Cryogenic Limited Model: PPM system, UK).

#### Aptamer binding on mMPs

The amine groups on the surface of the PDA@mMPs were first activated and then covalently coupled with amino-modified aptamers by sulpho-EDC/ NHS coupling method [53] (please see Step 3 in Fig. 1). The amine groups on the 5’ end of the aptamers were covalently attached to carboxylated mMPs by first solubilizing a quantity of 12.9 mg/mL of mMPs in 250 $\upmu$L of deionized water. Then, 100 mM of NSH and 20 mM of EDC were added to the solution to activate carboxyl groups. The mixture was incubated for 30 min by mixing at room temperature and washed with deionized water. The activated carboxyl groups were reacted with the amine label of aptamers by adding 1.0 $\upmu$L of 1.0 $\upmu$M aptamer solution in 250 $\upmu$L of PBS and incubating overnight.

### Bacteria isolation

Several bacterial cell suspensions were prepared either in PBS buffer (50 mM phosphate buffer, 150 mM NaCl; pH, 8.0) or blood samples. The procedure was adapted from previous reports [54, 55]. The aptamer-conjugated PDA@mMPs of 0.1 mg were mixed in a sample volume of 1.0 mL (which leads to 100 $\upmu$g/mL Apt@mMPs) with *S. pneumoniae* spiked blood samples. The mixture was vortexed to ensure full mixing and thereafter incubated at 25 ^∘^C with continuous shaking for 30 min. The collected cells were separated using magnetic pull-down after two rounds of washing with a combined volume of 2 mL of PBS. The cells that were separated and trapped on aptamer-magnetic beads were then used directly in triplicate experiments. The samples that were spiked with the target or non-target bacterial cells were combined with 0.1 mg of aptamer-nanoparticles, and then analyzed by culturing the samples on agar plates to determine the number of bacterial colonies. The eluted cells were subjected to serial dilutions, followed by plating on Tryptic Soy Broth (TSB) agar plates. Subsequently, the plates were incubated at 37 ^∘^C overnight. The viable colonies were quantified and reported as colony-forming units (CFU/mL).

The ratio of the percent captured cell was calculated as follows:

4 $$\begin{aligned} \text {Captured cells}\;[\%]= (C_c/C_t) \times 100 \end{aligned}$$

$C_t$ represents the bacteria concentration [CFU/mL] in the sample, while $C_c$ refers to the bacteria concentration computed as captured bacteria level.

**Fig. 2:**
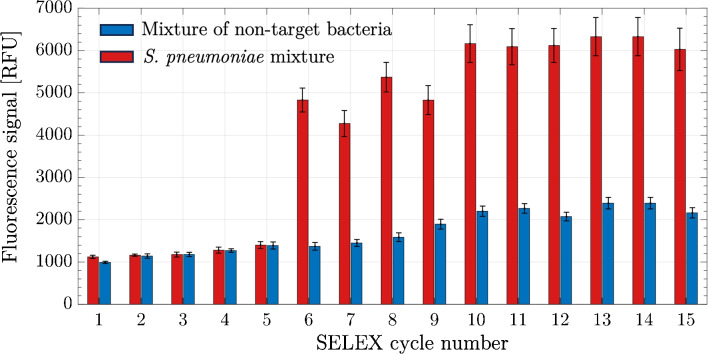
Library enrichment analysis. At the end of each SELEX cycle, the target binding amount was determined by the fluorescent labeling method. The target bacterial mixture consists of *S. pneumonia* serotypes, while the non-target bacterial mixture consists of *E. coli*, *S. aureus*, and *L. monocytogenes*. A total of 10^5^ total bacteria were used in the experiments. The values are the mean values with ± standard error from three independent repeated experiments

## Results and discussion

### Aptamer selection

The *S. pneumoniae* strains used in this study were carefully selected to represent the most common strains. *S. pneumoniae* has a significant degree of genetic variability and adaptability [56]. The virulence of *S. pneumoniae* is influenced by both serotype and genetic background [57]. The 7-valent PCV (pneumococcal conjugate vaccines) targeting strains are considered to be among causing the greatest disease burden. Thus, we chose serotypes 2, 4, 14, 6B, and 19F to select aptamers. DNA library members were amplified by PCR and used in SELEX cycles as single strands. The PCR products were checked to be at 79 bp after each cycle by agarose gel electrophoresis. A mixture of the *S. pneumoniae* strains ($2\times 10^5$ cells from each strain, $10^6$ cells in total) were used in the SELEX cycles for enrichment of target cell binding sequences.

Affinity enrichment was determined by detecting the increase in the amount of DNA sequences remaining bound to the target bacteria after each cycle (using the same amount of starting material). The improvement in the amount of binding indicates that the library complexity was reduced due to the increase in the proportion of sequences with only strong affinity. As seen in Fig. 2, the enrichment for the target bacteria started in the sixth cycle and continued at a high value in subsequent cycles. Similarly, there was no enrichment for the non-target bacteria. These affinity enrichment data reveal that SELEX selection was successful, and consequently, the candidate aptamer sequences were selected. At this stage, not all sequences with affinity were expected to show aptamer properties. Among the enriched sequences consisting of thousands of different sequences, the sequences with low specificity that bind to every surface would generally be in majority. For this reason, it was necessary to isolate DNA members from the last SELEX cycle, obtain DNA sequences by sequencing, and then determine aptamer sequences (sequences with specific and sensitive binding affinity) using bioinformatics methods. Here, the 15th cycle was selected for sequencing studies. While target bacterial binding was close to 6000 fluorescent signal (RFU), binding to non-target bacteria was around 2000 RFU, and there was an approximately threefold difference. These results indicated that the SELEX method successfully enriched specific sequences against *S. pneumoniae* cells.

**Fig. 3:**
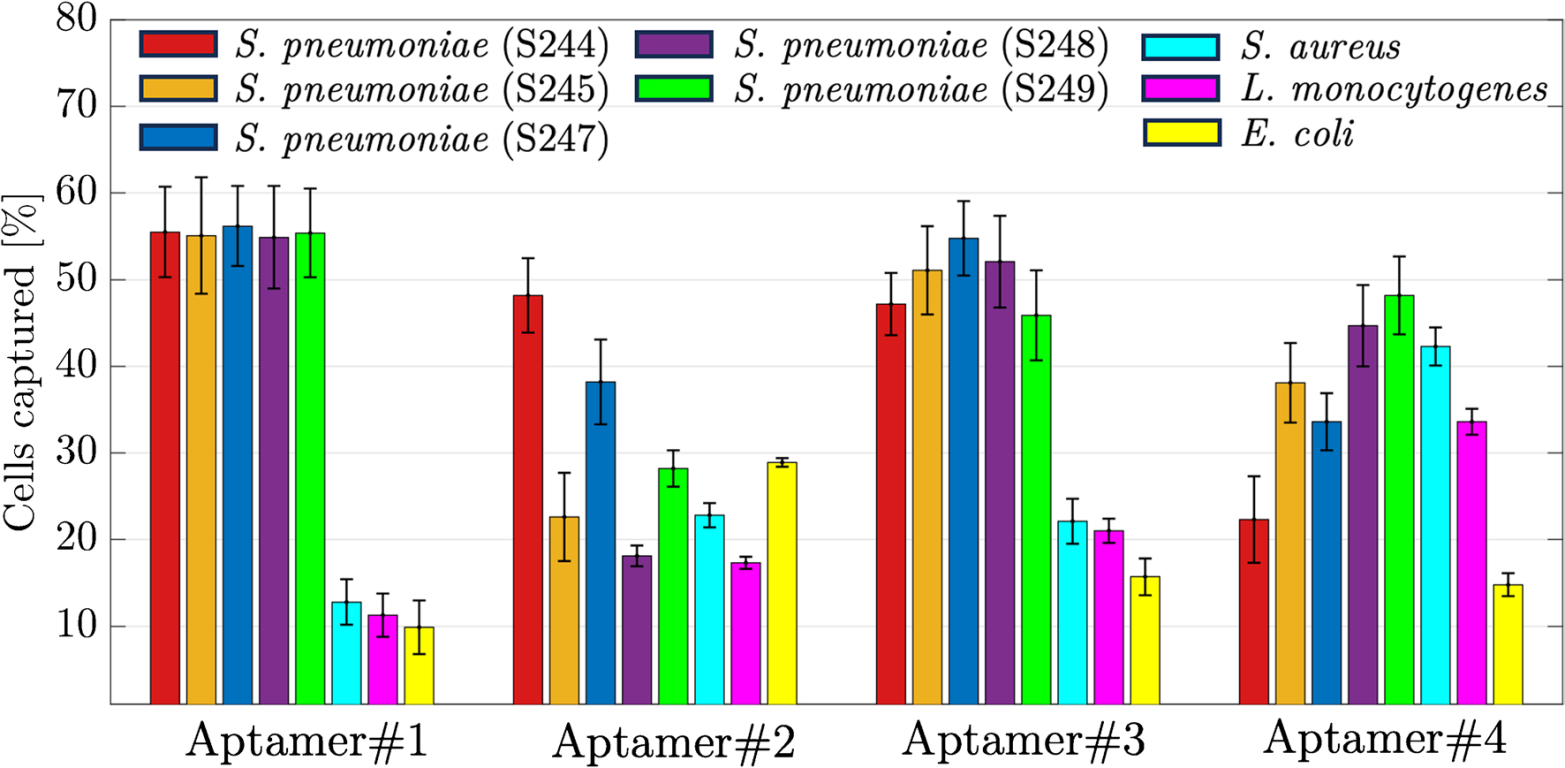
The comparison of capture efficiencies of aptamer candidates for target and non-target bacteria cells. In the assays, 10^4^ cells were mixed with 100 nM 5’-FAM-aptamer sequences in PBS, incubated for 30 min, and the captured cells were analyzed in flow cytometry. The values are the mean values with ± standard error from three independent repeated experiments

The PCR products obtained after the fifteenth cycle were subjected to further amplification using NGS forward and NGS reverse primers (please see Table 1), followed by NGS analysis. Upon completion of the sequencing process, 163,908 sequences were successfully sequenced, which were categorized into ten distinct groups based on the occurrence of motifs that were between six and seven bases in length. The first prevalent motif sequence, CTTCTM, was seen in a total of 121,431 sequences, accounting for almost 75% of the whole set of total sequences. The pattern AAGAAK was identified as the second most often occurring motif, with a total of 16,615 sequences containing it. The TCTCAR and CACKCR sequences were observed in 13,851 and 11,770 sequences, respectively. The motifs ranked second, third, and fourth in terms of frequency were seen in 10%, 9%, and 7% of the total number of sequences, respectively. Sequences that exhibited a high frequency of motifs were regarded as the most enriched sequences, indicating their higher affinity for target cells. In light of this rationale, the flow cytometry analysis was employed to investigate the binding affinities of a collection of aptamer candidates derived from the Motif#1, Motif#2, Motif#3, and Motif#4 groups which were the ones most frequently observed. The quantification of the affinities of these sequences, as shown in Table 3, towards 10^4^ *S. pneumoniae* cells was conducted using flow cytometry, using 5’-FAM labeling, as seen in Fig. 3.

**Fig. 4:**
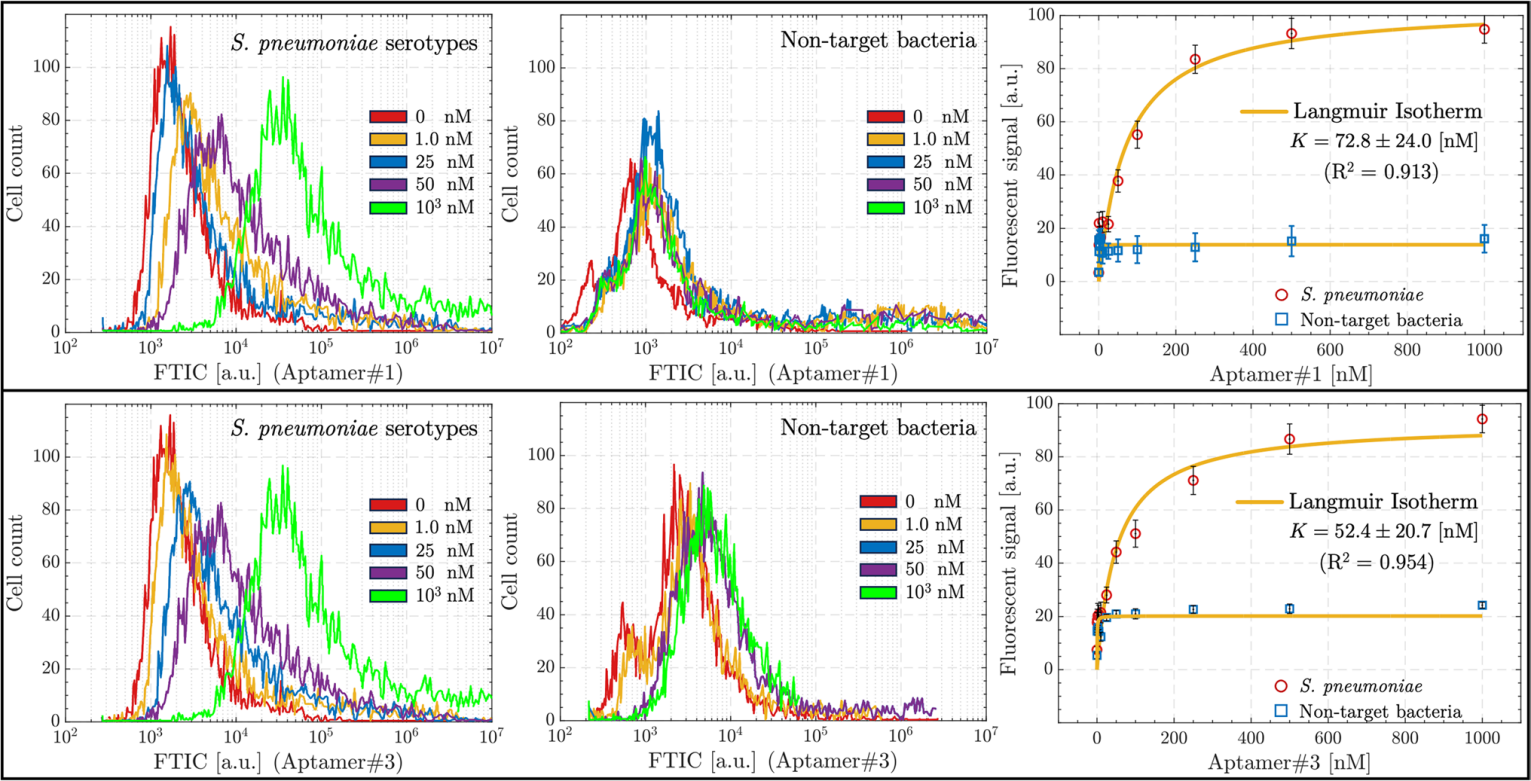
The binding analysis of the Aptamer#1 and Aptamer#3 sequenced by flow cytometry. Top row: Aptamer#1. Bottom Row: Aptamer#3. (From left to right) First: Mixture of *S. pneumoniae* serotypes (S244, S245, S247, S248, S249) samples of 10^4^ cells. Second: mixture of non-target bacteria (*S. aureus*, *L. monocytogenes*, *E. coli*) containing 10^4^ cells used as control. Third: Binding assays with various concentrations (the values are the mean values with ± standard error from three independent repeated experiments)

This study aimed to evaluate and compare the capture efficiency of several aptamer candidates for both target and non-target bacteria cells. During the experimental procedures, a total of 10^4^ cells were combined with 100 nM of 5’-FAM-aptamer sequences in PBS. This mixture was then incubated for a duration of 30 min, after which the captured cells were subjected to analysis using flow cytometry. The results revealed that Aptamer#1 have high specificity for *S. pneumoniae* isolates (244, 245, 247, 248, and 249) with mean capture efficiencies above 55%. In contrast, the aptamers demonstrated weak binding to non-target cells, with capture efficiencies below 13%. For Aptamer#2 with non-target cells, the capture assays showed 23.2% binding to *S. aureus*, 22.1% to *L. monocytogenes*, and 29.4% to *E. coli*. The binding of Aptamer#2 was varied for serotypes of *S. pneumoniae* (Fig. 3).

Since Aptamer#1 and Aptamer#3 sequences exhibited better specificity to *S. pneumoniae*, a detailed binding analysis for these two aptamer candidates was performed with samples spiked with 10^4^ CFU/mL cells in PBS. The binding experiments were performed with constant bacteria cell number with different aptamer concentrations. In cytometry analysis, it was observed that the binding ratio improved with increasing Aptamer#1 and Aptamer#3 concentrations (Fig. 4). The affinity constant to the *S. pneumoniae* isolate mixture was determined to be $72.8\pm 24.0$ nM for Aptamer#1, and $52.4\pm 20.7$ nM for Aptamer#3. Aptamer#1 specificity showed a maximum of 15% binding to control mixture bacteria. Aptamer#3 showed a maximum of 20% binding with slightly less specificity. According to these results, it was concluded that Aptamer#1 could be implemented in the magnetic purification system with a better performance.

**Fig. 5:**
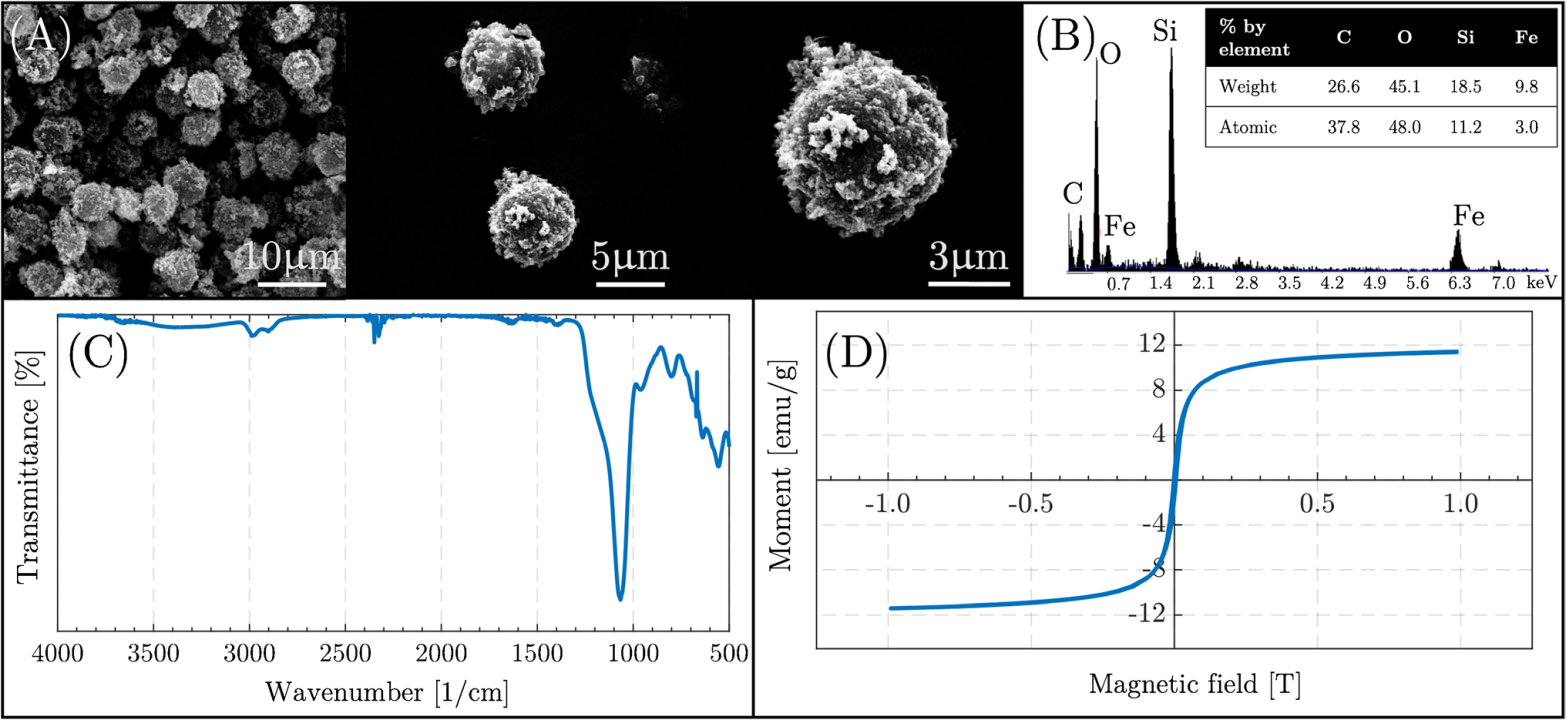
**A** Morphological structure (SEM imagesat 5000$\times$, 10,000$\times$, and 20,000$\times$ magnification with 10 $\upmu$m, 5 $\upmu$m, and 3 $\upmu$m scale bar, respectively), **B** surface chemistry (EDX results), **C** chemical structure (FTIR spectrum), and **D** magnetic properties (VSM results) of synthesized magnetic particles

### Characterization of mMPs

The particles were obtained in monodisperse around 6 $\upmu$m in size as seen in Fig. 5A. The carbon, oxygen, and iron content on the surface of the particle was determined *via* EDX. The organic and inorganic hybrid content was obtained around 26.6% C, 45.1% O, 18.5% Si, and 9.8% Fe as weight percentages, and 37.8% C, 48.0% O, 11.2% Si, and 3.0% Fe as atomic percentages as seen in Fig. 5B. The chemical structure of the particles was analyzed with FTIR. As shown in Fig. 5 B and C, the FTIR spectrum shows the inorganic part Fe_2_O_3_ characteristic peaks at 560 and 690 cm^-1^ [58]. The strong Si-O peak can be seen at 1010 cm^-1^. The organic part of the particles can be seen with a weak peak at 1390 cm^-1^ and 1630 cm^-1^ as amide groups of PDA [59], and C-H stretching at 2880 cm^-1^ and 2980 cm^-1^. The broad peak at 3500 cm^-1^ represents O-H and N-H functional groups of PDA coating on the particles’ surfaces. The magnetic property of the particles was analyzed by drawing a hysteresis curve as seen in Fig. 5D. The curve shows that the magnetic saturation value is around 10 emu/g and indicates that the mMPs exhibit superparamagnetic characteristics.

### Bacteria isolation

**Fig. 6:**
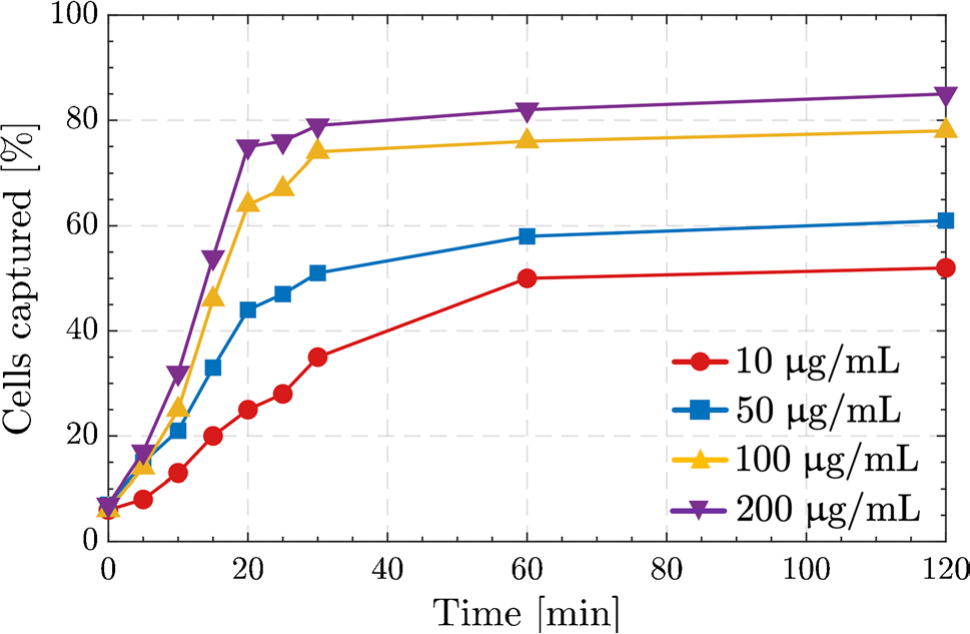
Captured *S. pneumoniae* with incubation time. Various Aptamer#1-conjugated mMPs (Apt@mMPs) at different concentrations (10, 50, 100, and 200 $\upmu$g/mL) were incubated with 10^3^ *S. pneumoniae* cells. The captured cell numbers were recorded according to incubation time

Bacterial capture tests were conducted utilizing Aptamer#1 binded PDA@mMPs (Apt@mMPs) in a PBS solution at 25^∘^C to optimize the experimental conditions for the quantity of mMPs and the duration of the incubation period. The immobilization efficiency was calculated as 71% from the difference of absorption spectra at 260 nm during aptamer binding procedure. Assuming 2 g/cm^-3^ density for PDA@mMPs, the number of immobilized aptamer molecules was calculated as 5.7$\times 10^4$ for each microparticle [60]. Figure 6 displays the capture percentages of *S. pneumoniae* cells when a constant cell number of 10^3^ CFU/mL is used over incubation durations of up to 2 h. As seen from the figure, the peak capture rates were achieved almost after 40 min for each mMPs concentration. Moreover, the higher the concentration of mMPs, the higher the capture efficiency; therefore, mMPs with a concentration of 200 $\upmu$g/mL have the best performance. However, the difference between 100 and 200 $\upmu$g/mL cases was not substantial; therefore, 100 $\upmu$g/mL was applied for bacteria isolation experiments. When the concentration of mMPs is above 50 $\upmu$g/mL, saturation was observed approximately after 20 min with a capture rate above 60%.

**Fig. 7:**
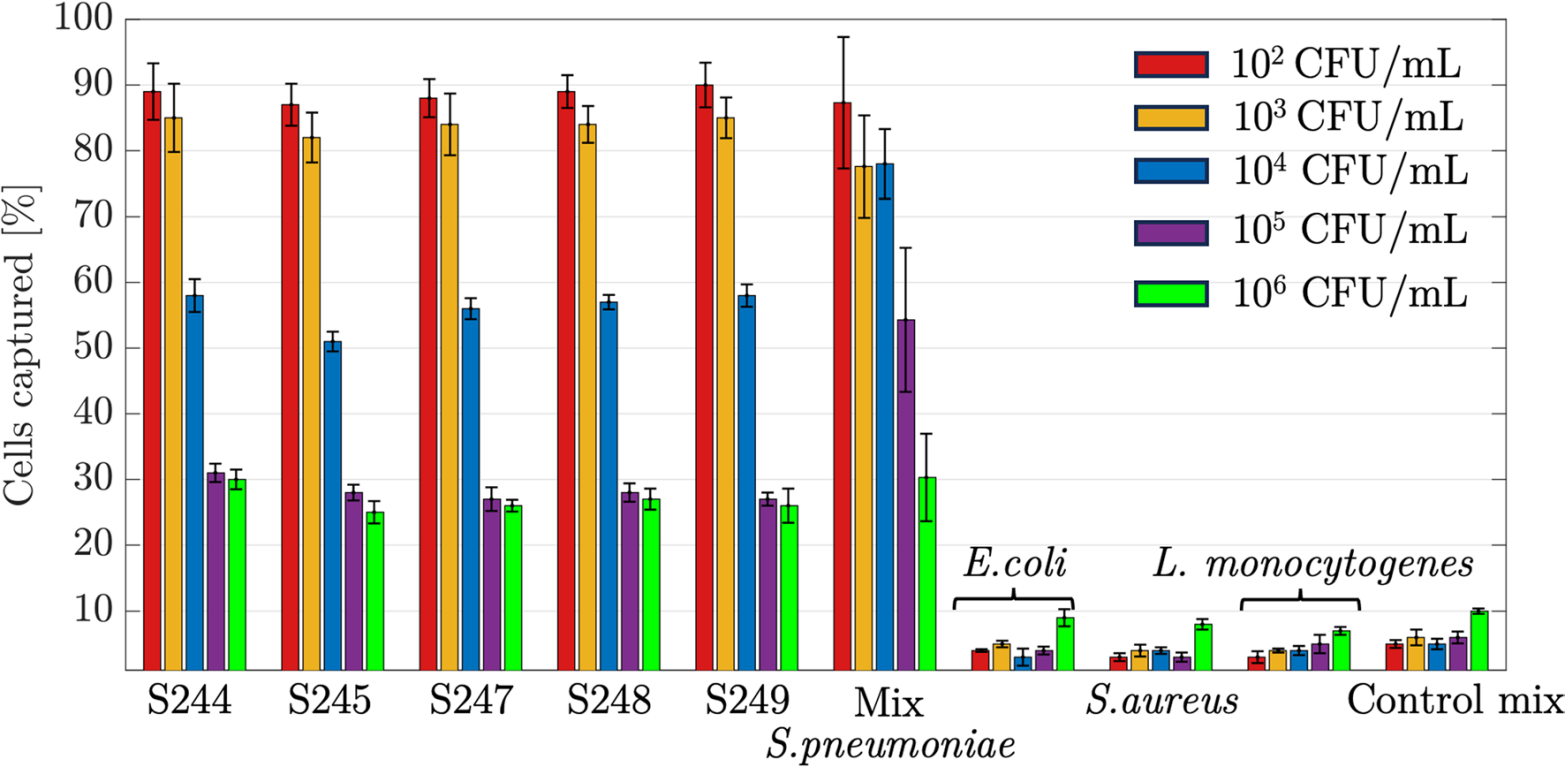
Captured *S. pneumoniae* at different cell concentrations (10^2^–10^6^ CFU/mL) in PBS. Five different serotypes of *S. pneumoniae* (S244, S245, S247, S248, S249) were tested individually and as a mixture, and *E. coli*, *S. aureus*, and *L. monocytogenes* were implemented as non-target bacteria. The values are the mean values with ± standard error from three independent repeated experiments

Figure 7 shows the capture percentages of *S. pneumoniae* cells by Apt@mMPs as investigated for samples spiked with concentrations ranging from 10^2^ to 10^6^ CFU/mL in PBS buffer. Five different serotypes of *S. pneumoniae* (S244, S245, S247, S248, S249) were tested individually, and as a mixture, and *E. coli*, *S. aureus*, and *L. monocytogenes* were implemented as non-target bacteria. For the *S. pneumoniae* serotypes, the capture percentages were highest at the lowest concentration (10^2^ CFU/mL), ranging from approximately 80 to 90%. As the concentration increased, the capture percentages generally reduced. At the highest concentration (10^6^ CFU/mL), the capture percentages ranged from 25 to 32%. The mixture of *S. pneumoniae* sample showed a similar trend, with a capture percentage of 88% at the lowest concentration and 32% at the highest concentration. In contrast, the non-target bacteria (*E. coli*, *S. aureus*, and *L. monocytogenes*) and their mixture showed consistently low capture percentages across all concentrations, ranging from 3 to 10%. These results suggest that the Apt@mMPs have a high specificity for *S. pneumoniae*, even in the presence of non-target bacteria. However, the efficiency of capture decreases as the concentration of *S. pneumoniae* increases. This could be due to the saturation of the aptamers on the PDA@mMPs which limits the number of *S. pneumoniae* cells that can be captured. Further optimization of the system may improve capture efficiency at higher concentrations. However, these results demonstrated that Apt@mMPs can be used for the isolation of predictable percentages of *S. pneumoniae*.

**Fig. 8:**
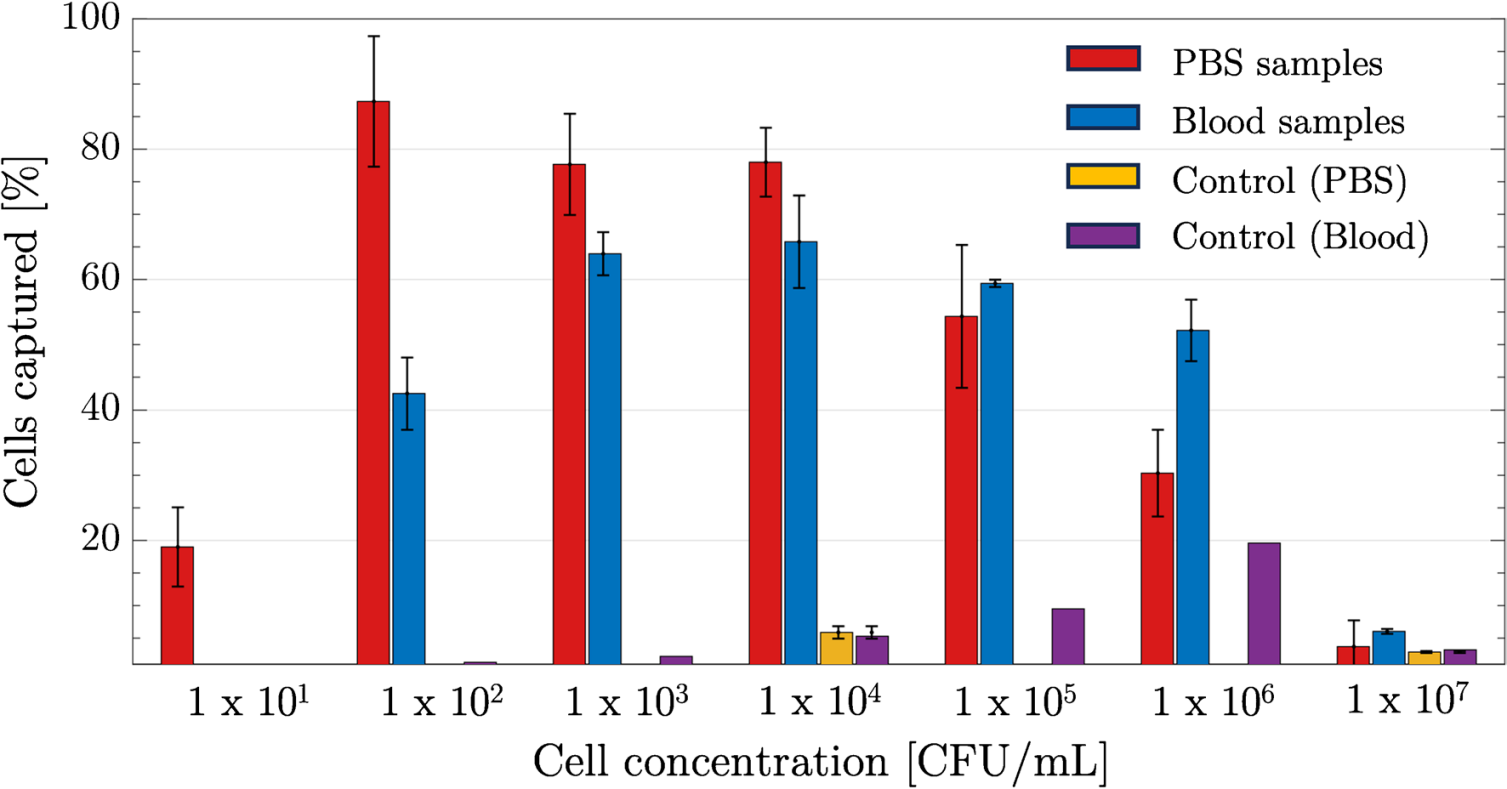
Capture experiments in bacteria-spiked PBS and blood samples (100 $\upmu$g Apt@mMPs were mixed with 1.0 mL samples for 30 min). The values are the mean values with ± standard error from three independent repeated experiments

The capture efficiencies of Apt@mMPs were evaluated for the capture of *S. pneumoniae* in PBS and blood samples with cell concentrations between 10^1^ and 10^7^ CFU/mL. The results are shown in Fig. 8. The capture efficiencies were slightly lower in blood samples than in PBS up to 10^4^ CFU/mL. The capture was significantly different for 10^2^ CFU/mL. For the cell concentration of 10^3^ CFU/mL, the cell captured values were very close to 74% and 64% for PBS and blood samples, respectively. Figure 8 also demonstrates the results for *S. aureus*, which serves as a control bacterium to demonstrate the specificity of our selected aptamers in capturing *S. pneumoniae*. The control samples with non-target bacteria showed very low capture efficiencies, indicating that the Apt@mMPs are specific for *S. pneumoniae*. By comparing the capture efficiency of *S. aureus* to that of *S. pneumoniae*, we can validate the selectivity of our assay system. At two different concentrations, namely 10^4^ and 10^7^ CFU/mL, the capture efficiency of the control bacteria, *S. aureus*, was observed to be approximately 6% and 3%, respectively. These results demonstrate that Apt@mMPs can be used reliably for sample preparation between 10^3^ and 10^5^ CFU/mL *S. pneumoniae* in blood samples.

## Concluding remarks

This study revealed the novel aptamer series *via* targeting *S. pneumoniae* bacteria cells with Cell-SELEX methodology. The results demonstrated that aptamer-conjugated mMPs can be used for the efficient capture of *S. pneumoniae* bacteria cells in both PBS buffer and blood samples. The rate of cell captured was high and specific, with low capture of non-target bacteria such as *E. coli*, *S. aureus*, and *L. monocytogenes*. These results suggest that Apt@mMPs have the potential to be used for the development of rapid and sensitive diagnostic tests for *S. pneumoniae* infections. Aptamers have documented advantages compared to other ligands such as enhanced shelf life, stability for extreme physical conditions, and machine-synthesis of the ligand [20, 61, 62]. This methodology also proves that the aptamers could be selectively implemented for biosensing, capturing, and targeting systems with combination of micro/nanoparticles. In further studies, the Apt@mMPs may be potentially employed for point-of-care testing, capturing other biological substances such as exosomes, viruses, cancer cells, or other bacteria in batch or microfluidic systems. In addition, various detection systems may be also utilized with Apt@mMPs such as SERS, SPR, or other spectroscopic methods by changing the functionalization of microparticles *via* fluorescent molecules, silver nanoparticles, or gold nanoparticles. Indeed, this study is the first step towards the isolation of *S. pneumoniae* on a novel 3D-printed magnetic microfluidic platform [40] for rapid detection [41]. The microparticle synthesis may be also integrated in a microfluidic platform for a better monodispersity [48, 63, 64].

## Data Availability

The data set used and analyzed during the current study are available from the corresponding author on reasonable request.

## References

[1] Talebi Bezmin Abadi A, Rizvanov AA, Haertlé T, Blatt NL (2019). World health organization report: current crisis of antibiotic resistance. BioNanoScience.

[2] Ikuta KS, Swetschinski LR, Aguilar GR, Sharara F, Mestrovic T, Gray AP, Weaver ND, Wool EE, Han C, Hayoon AG (2022). Global mortality associated with 33 bacterial pathogens in 2019: a systematic analysis for the Global Burden of Disease Study 2019. Lancet.

[3] Lynch JP, Zhanel GG (2009). Streptococcus pneumoniae: epidemiology, risk factors, and strategies for prevention. Semin Respir Crit Care Med.

[4] Bar-Zeev N, Swarthout TD, Everett DB, Alaerts M, Msefula J, Brown C, Bilima S, Mallewa J, King C, von Gottberg A (2021). Impact and effectiveness of 13-valent pneumococcal conjugate vaccine on population incidence of vaccine and non-vaccine serotype invasive pneumococcal disease in Blantyre, Malawi, 2006–18: prospective observational time-series and case-control studies. Lancet Glob Health.

[5] Zhang Y, Isaacman DJ, Wadowsky RM, Rydquist-White J, Post JC, Ehrlich GD (1995). Detection of Streptococcus pneumoniae in whole blood by PCR. J Clin Microbiol.

[6] Weng C-C, Chao C-Y, Wu S-T, Tsou P-H, Chen W-T, Li B-R, Li Y-K (2021). Integration of Ni/NiO nanoparticles and a microfluidic ELISA chip to generate a sensing platform for Streptococcus pneumoniae detection. RSC Adv.

[7] Sande MG, Çaykara T, Silva CJ, Rodrigues LR (2020). New solutions to capture and enrich bacteria from complex samples. Med Microbiol Immunol.

[8] Düven G, Çetin B, Kurtuldu H, Gündüz GT, Tavman Ş, Kışla D (2019). A portable microfluidic platform for rapid determination of microbial load and somatic cell count in milk. Biomed Microdev.

[9] Garrido-Jareño M, Puchades-Carrasco L, Orti-Pérez L, Sahuquillo-Arce JM, del Carmen Meyer-García M, Mollar-Maseres J, Lloret-Sos C, Gil-Brusola A, López-Hontangas JL, Beltrán-Garrido JM (2021). A surface plasmon resonance based approach for measuring response to pneumococcal vaccine. Sci Rep.

[10] Singhal C, Bruno JG, Kaushal A, Sharma TK (2021). Recent advances and a roadmap to aptamer-based sensors for bloodstream infections. ACS Appl Bio Mater.

[11] Caflisch KM, Suh GA, Patel R (2019). Biological challenges of phage therapy and proposed solutions: a literature review. Expert Rev Anti Infect Ther.

[12] Duperthuy M (2020). Antimicrobial peptides: virulence and resistance modulation in Gram-negative bacteria. Microorganisms.

[13] Wang H, Ma Z, Qin J, Shen Z, Liu Q, Chen X, Wang H, An Z, Liu W, Li M (2019). A versatile loop-mediated isothermal amplification microchip platform for streptococcus pneumoniae and mycoplasma pneumoniae testing at the point of care. Biosens Bioelectron.

[14] Çetin B, Özer MB, Solmaz ME (2014). Microfluidic bio-particle manipulation for biotechnology. Biochem Eng J.

[15] Beyor N, Seo TS, Liu P, Mathies RA (2008). Immunomagnetic bead-based cell concentration microdevice for dilute pathogen detection. Biomed Microdevices.

[16] Kwon Y, Hara CA, Knize MG, Hwang MH, Venkateswaran KS, Wheeler EK, Bell PM, Renzi RF, Fruetel JA, Bailey CG (2008). Magnetic bead based immunoassay for autonomous detection of toxins. Anal Chem.

[17] Choi J-W, Liakopoulos TM, Ahn CH (2001). An on-chip magnetic bead separator using spiral electromagnets with semi-encapsulated permalloy. Biosens Bioelectron.

[18] Guo S, Deng Y, Zhao L, Chan H, Zhao X (2008). Effect of patterned micro-magnets on superparamagnetic beads in microchannels. J Phys D Appl Phys.

[19] Modh H, Scheper T, Walter J-G (2018). Aptamer-modified magnetic beads in biosensing. Sensors.

[20] Mairal T, Özalp VC, Lozano Sánchez P, Mir M, Katakis I, O’Sullivan CK (2008). Aptamers: molecular tools for analytical applications. Anal Bioanal Chem.

[21] Xu H, Mao X, Zeng Q, Wang S, Kawde A-N, Liu G (2009). Aptamer-functionalized gold nanoparticles as probes in a dry-reagent strip biosensor for protein analysis. Anal Chem.

[22] Zhou Y, Lv S, Wang X-Y, Kong L, Bi S (2022). Biometric photoelectrochemical-visual multimodal biosensor based on 3D hollow HCdS@Au nanospheres coupled with target-induced ion exchange reaction for antigen detection. Anal Chem.

[23] Zhi S, Zhang X, Zhang J, Wang X-Y, Bi S (2023). Functional nucleic acids-engineered bio-barcode nanoplatforms for targeted synergistic therapy of multidrug-resistant cancer. ACS Nano.

[24] Hai X, Ji M, Yu K, Tian T, Cui Z, Bi S, Zhang X (2023). Acid-responsive DNA-Au nanomachine with active/passive dual-targeting capacity for combinational cancer therapy. Materials Today Nano.

[25] Wang C-H, Wu J-J, Lee G-B (2019). Screening of highly-specific aptamers and their applications in paper-based microfluidic chips for rapid diagnosis of multiple bacteria. Sens Actuators, B Chem.

[26] Su Y, Zhu L, Wu Y, Liu Z, Xu W (2022) Progress and challenges in bacterial whole-cell-components aptamer advanced screening and site identification. TrAC Trends Anal Chem 116731

[27] Sefah K, Shangguan D, Xiong X, O’donoghue MB, Tan, W (2010) Development of DNA aptamers using Cell-SELEX. Nat Protoc 5(6):1169–1185 10.1038/nprot.2010.66 20539292

[28] Kolm C, Cervenka I, Aschl UJ, Baumann N, Jakwerth S, Krska R, Mach RL, Sommer R, DeRosa MC, Kirschner AK (2020). DNA aptamers against bacterial cells can be efficiently selected by a SELEX process using state-of-the art QPCR and ultra-deep sequencing. Sci Rep.

[29] Zhu C, Feng Z, Qin H, Chen L, Yan M, Li L, Qu F (2023) Recent progress of SELEX methods for screening nucleic acid aptamers. Talanta 124998 10.1016/j.talanta.2023.124998 37527564

[30] Yu F, Chen J, Wang Z, Yang H, Li H, Jia W, Xue S, Xie H, Xu D (2021). Screening aptamers for serine $$\beta$$-lactamase-expressing bacteria with Precision-SELEX. Talanta.

[31] Nikam PS, Palachandra S, Kingston JJ (2022). In vitro selection and characterization of ssDNA aptamers by cross-over SELEX and its application for detection of S. Typhimurium Anal Biochem.

[32] Amraee M , Oloomi M, Yavari A, Bouzari S (2017). DNA aptamer identification and characterization for E. coli o157 detection using cell based SELEX method. Anal Biochem.

[33] de Melo MIA, da Silva Cunha P, Ferreira IM, de Andrade ASR (2023). DNA aptamers selection for Staphylococcus aureus cells by SELEX and Cell-SELEX. Mol Biol Rep.

[34] Zhou Z, Lan X, Zhu L, Zhang Y, Chen K, Zhang W, Xu W (2023). Portable dual-aptamer microfluidic chip biosensor for bacillus cereus based on aptamer tailoring and dumbbell-shaped probes. J Hazard Mater.

[35] Kusumawati A, Mustopa AZ, Wibawan IWT, Setiyono A, Sudarwanto MB (2022). A sequential toggle cell-SELEX DNA aptamer for targeting Staphylococcus aureus, Streptococcus agalactiae, and Escherichia coli bacteria. J Genet Eng Biotechnol.

[36] Zhang Y, Xing H, Bolotnikov G, Krämer M, Gotzmann N, Knippschild U, Kissmann A-K, Rosenau F (2023). Enriched aptamer libraries in fluorescence-based assays for Rikenella microfusus-specific gut microbiome analyses. Microorganisms.

[37] Duan Y, Zhang C, Wang Y, Chen G (2022). Research progress of whole-cell-SELEX selection and the application of cell-targeting aptamer. Mol Biol Rep.

[38] Schmitz FRW, Valério A, de Oliveira D, Hotza D (2020). An overview and future prospects on aptamers for food safety. Appl Microbiol Biotechnol.

[39] Hatipoğlu U, Kibar G, Çetin B (2019) Enrichment of samples inside microchannels by using magnetic particles. Int Patent Appl No: PCT/TR2019/050728 https://patents.google.com/patent/WO2020050809A2/en

[40] Kibar G, Sarıarslan B, Doganay S, Yıldız G, Usta B, Çetin B (2024). Novel 3D-printed microfluidic magnetic platform for rapid DNA isolation. Anal Chem.

[41] Babaie Z (2023) Magnetic microfluidic platform for bacteria isolation and detection. Master’s thesis, Bilkent University, Ankara, Türkiye

[42] Dursun AD, Borsa BA, Bayramoglu G, Arica MY, Ozalp VC (2022). Surface plasmon resonance aptasensor for brucella detection in milk. Talanta.

[43] Hulme EC, Trevethick MA (2010). Ligand binding assays at equilibrium: validation and interpretation. British J Pharmacol.

[44] Cleary DW, Jones J, Gladstone RA, Osman KL, Devine VT, Jefferies JM, Bentley SD, Faust SN, Clarke SC (2022). Changes in serotype prevalence of streptococcus pneumoniae in Southampton, UK between 2006 and 2018. Sci Rep.

[45] Ersoy Omeroglu E, Sudagidan M, Yurt MNZ, Tasbasi BB, Acar EE, Ozalp VC (2021). Microbial community of soda lake van as obtained from direct and enriched water, sediment and fish samples. Sci Rep.

[46] Bailey TL, Boden M, Buske FA, Frith M, Grant CE, Clementi L, Ren J, Li WW, Noble WS (2009) Meme suite: tools for motif discovery and searching. Nucleic Acids Res. 37(suppl_2):202–208 10.1093/nar/gkp335 PMC2703892 19458158

[47] Liu D, Hu B, Peng D, Lu S, Gao S, Li Z, Wang L, Jiao B (2020). Isolation ssDNA aptamers specific for both live and viable but nonculturable state Vibrio vulnificus using whole bacteria-SEILEX technology. RSC Adv.

[48] Kibar G, Çalışkan U, Erdem EY, Çetin B (2019). One-pot synthesis of organic-inorganic hybrid polyhedral oligomeric silsesquioxane microparticles in a double-zone temperature controlled microfluidic reactor. J Polymer Sci A Polymer Chem.

[49] Kibar G (2023). Effect of crosslinking agent on mesoporous spherical POSS hybrid particles: synthesis, characterization and thermal stability. J Inorg Organomet Polym Mater.

[50] Kibar G, Tuncel A (2018). Gold-nanoparticle decorated monosized magnetic polymer based catalyst: reduction of 4-nitrophenol. J Inorg Organomet Polym Mater.

[51] Günal G, Kip Ç, Öğüt SE, Usta DD, Şenlik E, Kibar G, Tuncel A (2017). Human genomic DNA isolation from whole blood using a simple microfluidic system with silica-and polymer-based stationary phases. Mater Sci Eng C.

[52] Kibar G, Dinç DŞÖ (2019). In-situ growth of Ag on mussel-inspired polydopamine@ poly (M-POSS) hybrid nanoparticles and their catalytic activity. J Environ Chem Eng.

[53] Odeh F, Nsairat H, Alshaer W, Ismail MA, Esawi E, Qaqish B, Bawab AA, Ismail SI (2019). Aptamers chemistry: chemical modifications and conjugation strategies. Molecules.

[54] Ozalp VC, Bayramoglu G, Kavruk M, Keskin BB, Oktem HA, Arica MY (2014). Pathogen detection by core-shell type aptamer-magnetic preconcentration coupled to real-time PCR. Anal Biochem.

[55] Borsa BA, Tuna BG, Hernandez FJ, Hernandez LI, Bayramoglu G, Arica MY, Ozalp VC (2016). Staphylococcus aureus detection in blood samples by silica nanoparticle-oligonucleotides conjugates. Biosens Bioelectron.

[56] Kim JO, Weiser JN (1998). Association of intrastrain phase variation in quantity of capsular polysaccharide and teichoic acid with the virulence of Streptococcus pneumoniae. J Infect Dis.

[57] McAllister LJ, Ogunniyi AD, Stroeher UH, Leach AJ, Paton JC (2011). Contribution of serotype and genetic background to virulence of serotype 3 and serogroup 11 pneumococcal isolates. Infect Immun.

[58] Jafari A, Farjami Shayesteh S, Salouti M, Boustani K (2015). Dependence of structural phase transition and lattice strain of Fe 3 o 4 nanoparticles on calcination temperature. Ind J Phys.

[59] Fu J, Chen Z, Wang M, Liu S, Zhang J, Zhang J, Han R, Xu Q (2015). Adsorption of methylene blue by a high-efficiency adsorbent (polydopamine microspheres): kinetics, isotherm, thermodynamics and mechanism analysis. Chem Eng J.

[60] Hernandez FJ, Hernandez LI, Pinto A, Schäfer T, Özalp VC (2013). Targeting cancer cells with controlled release nanocapsules based on a single aptamer. Chem Comm.

[61] Thiviyanathan V, Gorenstein DG (2012). Aptamers and the next generation of diagnostic reagents. PROTEOMICS-Clin Appl.

[62] Sequeira-Antunes B, Ferreira HA (2023). Nucleic acid aptamer-based biosensors: a review. Biomedicines.

[63] Aşık MD, Kaplan M, Çetin B, Sağlam N (2021). Synthesis of iron oxide core chitosan nanoparticles in a 3D printed microfluidic device. J Nanopart Res.

[64] Kibar G, Şahinoğlu OB, Kılınç B, Erdem EY, Çetin B, Özalp CV (2024). Biosensor for ATP detection via aptamer modified PDA@POSS nanoparticles synthesized in a microfluidic reactor. Microchim Acta.

